# “From hero to villain” tracheovascular fistula as an unusual complication of a silicone stent in tracheal stenosis due to brachiocephalic trunk aneurysm

**DOI:** 10.1016/j.rmcr.2025.102349

**Published:** 2025-12-24

**Authors:** Olivia Sánchez-Cabral, Alan Jhunior Solis, Patzy Marcela Leal-Valenzuela, María Fernanda Negrete-García, Hazel Vázquez-Rojas

**Affiliations:** Bronchoscopy and Interventional Pneumology Unit of the National Institute of Respiratory Diseases (INER), Mexico

**Keywords:** Tracheal stenosis, Brachiocephalic trunk aneurysm, Silicone stent, Tracheovascular fistula

## Abstract

Aortic and great vessel aneurysms may cause stenosis of adjacent structures, including the airway, which can be fatal if severe and may require advanced airway management.

We report a case of tracheal stenosis secondary to a brachiocephalic aneurysm that initially responded to silicone stent placement but later developed a tracheal fistula into the aneurysm. Definitive surgical repair was achieved after stent removal. This potential complication should be considered in the management of such cases.

## 1 Introduction

Airway obstruction is a rare but potentially life-threatening complication of large vessel aneurysms such as the aorta [1].

We report a case of a fistula between the trachea and brachycephalic trunk in relation to a silicone stent. The computed tomography (CT) images from the patient's diagnosis have already been reported [2].

## 2 Case report

**Fig. 1:**
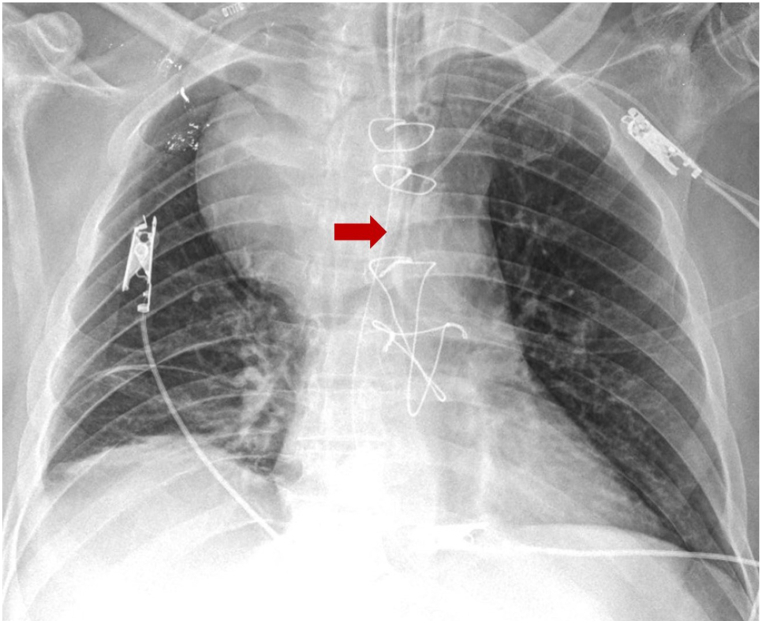
Antero-posterior chest x-ray: Giant aneurysm of the brachiocephalic trunk that conditions severe extrinsic compression of the distal trachea (red arrow). Orotracheal tube in proximal trachea.

Male, 56 years old, hypertension and type 2 diabetes mellitus history, presented with dry cough, dysphonia, and progressive dysphagia, admitted at emergency department due to stridor, which required orotracheal intubation due to respiratory failure (Fig. 1). Chest CT showed a partially thrombosed saccular aneurysm of the branchicephalic trunk of 8 cm × 7.2 cm, causing severe extrinsic compression of the distal trachea and adjacent structures [2]. An emergency surgery was performed with the right carotid-carotid and brachiocephalic-subclavian bypasses on the right side and, the partially thrombosed aneurysm was left for a second treatment.

After 24 hours the patient was extubated but developed tachypnea and stridor. The patient was intolerant to a non-invasive mechanical ventilation, therefore it was decided to re-intubate him orotracheally. Following another failed extubation attempts an interventional pulmonology consultation from our unit was requested.

**Fig. 2:**
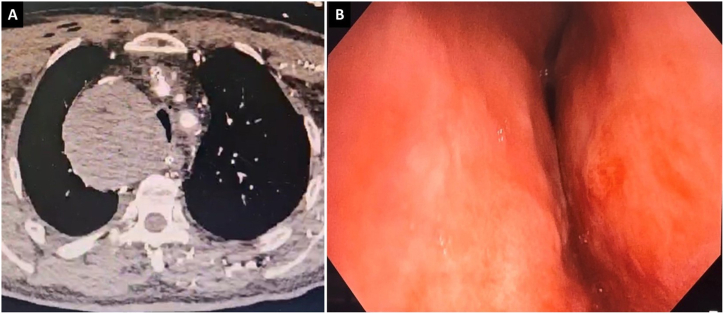
A: Chest CT scan, axial section: extrinsic compression of the trachea. B: Flexible bronchoscopy: severe extrinsic compression of the distal trachea at the expense of the right lateral wall.

**Fig. 3:**
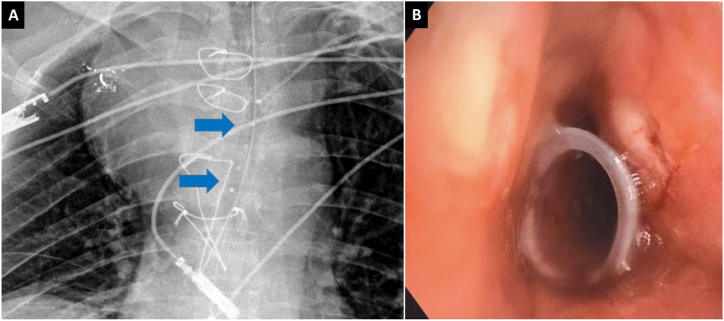
A: Chest X-ray after silicone stent placement in the distal trachea, radiopaque stent buttons (blue arrows) are observed. B: Bronchoscopy: the proximal end of the silicone stent is observed in the middle trachea.

The orotracheal tube was removed and patient was inmmediately intubated using a rigid tracheoscope number 14 (RB KARL STORZ® Tuttlingen, Germany). A checkup performed with a flexible bronchoscope (BF-1TH190 (Olympus, Europe, Germany) revealed an extrinsic stenosis of the mid and distal trachea due to extrinsic compression of >90 %, measuring 4cm in length up to the main carina (Fig. 2). Subsequently, a Dumond® 15 × 50 mm silicone stent (Novatech, La Ciotat, France) was placed with a rigid introducer at 5 mm from the main carina, leaving the tracheal lumen and both main bronchi permeable (Fig. 3). Twelve hours later the patient was successfully extubated and discharged home a few days later.

**Fig. 4:**
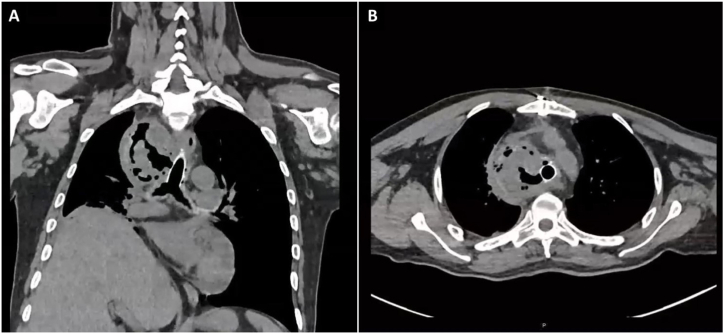
Frontal section computed tomography (A) and axial section (B): partially thrombosed brachiocephalic trunk aneurysm with hydroaeric level and trachea fistulization contained by silicone stent wall was observed.

**Fig. 5:**
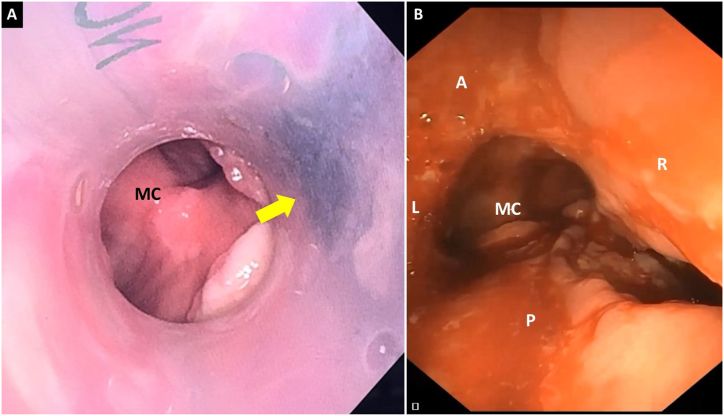
Bronchoscopy: A: blackish image is observed through the wall of the silicone stent (yellow arrow) in relation to necrosis and fistulization of the tracheal wall to the brachiocephalic trunk aneurysm. B: Distal trachea after stent removal, 2 cm lesion is observed on the right lateral wall of the trachea. MC: main carina, A: anterior, P: posterior, L: Left, R: right.

One week later, a bronchoscopy followed-up was performed, the stent was completely permeable. However, 4 weeks after stent placement, the patient was admitted into the emergency room with fever; chest CT showed a thrombosed saccular aneurysm and probable tracheovascular fistula (Fig. 4). Given this suspected diagnosis, the patient was connected to a veno-venous extracorporeal membrane oxygenation (ECMO V-V); flexible bronchoscopy was performed. An image suggestive of a tracheovascular fistula was observed on the right side wall of the stent. The stent was removed through the rigid tracheoscope, a continuity defect connecting the trachea to the aneurismal sac of approximately 2 cm in length was observed (Fig. 5).

The injury was surgically repaired, and the aneurysm was completely excluded by the Cardiothoracic Surgery Department. A week later the ECMO-VV was disconnected and the patient was discharged home a few days later.

## 3 Discussion

Airway compression due to vascular pathologies is rare and can be congenital, such as double aortic arch, aberrant subclavian artery or Kommerell's diverticulum, or acquired, such as aneurysm or aortic dissection [1,3].

Extrinsic tracheobronchial compression in mild cases is usually asymptomatic; but if the compression is significant, patients may experience dyspnea, cough, stridor or dysphagia if the esophagus is compressed; if there is aortic dissection, chest pain may also occur [1,4]. Diagnosis is confirmed with CT and bronchoscopy checkup [3], as was our patient case.

When airway compression is severe and life-threatening, immediate orotracheal intubation treatment as a bypass therapy is necessary to maintain airway patency until the vascular disorder is repair [4]. At this point, airway stents may also be an alternative if the patient cannot be extubated or there is no improvement in airway after surgical repair [1,[3], [4], [5]].

Tracheobronchial stents can save lives by allowing respiratory recovery and airway patency in the short and long term. Unresectable malignant neoplasms is the main indications. Stents are also used in stenosis due to benign conditions. When silicone stents are preferred, they are placed folded using a rigid inserter and bronchoscope [6]. Uncoated self-expandable metallic stents (SEMS) are not recommended to be used for benign airway stenosis [7], in fact, the Food and Drug Administration (FDA) advised against the use of these stents in benign stenoses in 2005 [8].

In the documented case, the patient continued to have tracheal stenosis despite surgery. A few minutes after attempt to extubate the patient stridor appeared, therefore, an orotracheal reintubation was required. Upon a multidisciplinary evaluation, to place a tracheal stent was decided. A silicone stent was chosen due to its availability at in our center at that time and because this type of stent is recommended for benign stenosis [6]. The response was spectacular and extubation was quickly achieved.

Literature on airway stenting for tracheobronchial compression due to aortic and great vessel aneurysms is limited, with most reports describing cases which SEMS were primarily used [4,[9], [10], [11], [12], [13], [14], [15], [16], [17], [18]], less frequently silicone stents [5,19] and in some cases of both types [1,9,20].

Silicone stents have the advantage of generating little granulation tissue, but they carry a higher migration risk, especially when caused by benign stenoses with a purely extrinsic component; they also carry a higher risk of mucous plugging and bacterial colonization [1,6,9]; however, airway wall perforation and bronchovascular fistulas are rare [21].

Bronchial or tracheo-aortic/vascular fistulas usually begin with hemoptysis, which can be fatal if massive [10,12,17]. In this case hemoptysis was not shown, the patient just had fever. This could be because necrosis point and fistula formation in the tracheal wall was retained by the silicone stent, unlike previous reported cases where hemoptysis began where SEMS were placed. Only three cases of aortobronchial fistulas in airway stenosis due to aortic aneurysms related to SEMS were found; two with Gianturco stent [12,17] and one with Ultraflex stent [10]. Of these, only one case survived after surgical repair [17]. No reports of tracheal fistula with brachiocephalic trunk related to silicone stent were found, therefore, this is the first reported case.

In the reported case, formation of the fistula was probably caused by radial force exerted by both the aneurysm and the stent itself on the tracheal wall. This could have caused microvasculature of the tracheal wall ischemia, with subsequent necrosis and fistula formation, which was stopped by the stent starting only with fever. Fortunately, the patient survived after ECMO-VV connection and surgical repair involving a multidisciplinary team.

In this context, customizing stents with 3D printed could reduce the risk of these complications by adapting to anatomical variations and better mimicking the physiological conditions of the airway [6]. Unfortunately, this technology is not yet available in our center.

## 4 Conclusion

Vascular pathologies as a cause of benign extrinsic airway stenosis shall be considered, where stents can be an alternative as a bridge therapy to correct vascular abnormalities. Radial strength of the stents must be considered, and tracheovascular fistula must be monitored as a possible complication. It is necessary to develop new stents with better design and materials to reduce the risk of these complications.

## CRediT authorship contribution statement

**Olivia Sánchez-Cabral:** Writing – review & editing, Writing – original draft, Visualization, Validation, Supervision, Project administration, Methodology, Investigation, Data curation, Conceptualization. **Alan Jhunior Solis:** Writing – review & editing, Writing – original draft, Visualization, Validation, Supervision, Software, Resources, Project administration, Methodology, Investigation, Formal analysis, Data curation, Conceptualization. **Patzy Marcela Leal-Valenzuela:** Writing – review & editing, Writing – original draft, Visualization, Validation, Software, Resources, Methodology, Investigation, Data curation, Conceptualization. **María Fernanda Negrete-García:** Writing – review & editing, Writing – original draft, Visualization, Validation, Resources, Methodology, Investigation, Data curation, Conceptualization. **Hazel Vázquez-Rojas:** Writing – review & editing, Writing – original draft, Visualization, Validation, Methodology, Investigation, Formal analysis, Data curation, Conceptualization.

## Declaration of competing interest

The authors declare that they have no known competing financial interests or personal relationships that could have appeared to influence the work reported in this paper.
